# Variation Among Grain Elevator Testing Sites and Analytical Cross-Reactivity of Commercial Immunoassay Kits for Deoxynivalenol Detection in Maize

**DOI:** 10.3390/toxins18020081

**Published:** 2026-02-04

**Authors:** Beatrice Gedion, Victor Limay-Rios, J. David Miller, David C. Hooker, Arthur W. Schaafsma

**Affiliations:** 1 Department of Plant Agriculture, University of Guelph, Ridgetown Campus, Ridgetown, ON N0P 2C0, Canada; beatrice.gedion@corteva.com (B.G.); vlimay@gmail.com (V.L.-R.); dhooker@uoguelph.ca (D.C.H.); 2 Department of Chemistry, Carleton University, Ottawa, ON K1S 5B6, Canada; davidmiller@cunet.carleton.ca

**Keywords:** deoxynivalenol, *Fusarium graminearum*, immunoassay, lateral flow device, ELISA, cross-reactivity, grain elevators, LC–MS/MS

## Abstract

Commercial immunoassay-based test kits are widely used for rapid screening of deoxynivalenol (DON) in maize; however, inconsistent results are frequently observed under commercial testing conditions. This study evaluated two distinct contributors to such variability: analytical cross-reactivity of commercial DON immunoassays and between-site variability arising from routine grain elevator testing practices. Under controlled laboratory conditions, all kits accurately measured DON but responded differently to co-occurring DON derivatives. In naturally contaminated maize, immunoassay results reflected the combined presence of DON and co-occurring derivatives, consistent with differences in antibody specificity. An interlaboratory comparison involving multiple grain elevators analyzing identical blinded samples demonstrated substantial between-site variability in reported DON concentrations, with about 16% of results deviating by more than ±20% from the LC–MS/MS reference value. Collectively, these findings show that inconsistent DON test outcomes arise from the combined effects of antibody cross-reactivity and site-specific testing variability, rather than from any unreliability of the analytical methods themselves. This finding highlights the importance of interpreting rapid DON measurements considering these factors.

## 1. Introduction

During the 2018 growing season, Ontario, Canada, experienced a severe Fusarium epidemic in maize. Deoxynivalenol (DON) contamination was widespread and disrupted grain delivery across the province. Industry stakeholders widely observed inconsistent DON test results at delivery. Identical grain loads often produced different values across locations or even on repeat testing at the same elevator. This situation eroded stakeholder confidence and raised questions about the reliability of rapid DON testing under commercial conditions.

Deoxynivalenol (DON) is among the most frequently detected Fusarium mycotoxins in maize and other cereal grains grown in temperate regions [1,2,3,4,5]. Its occurrence is driven by interactions among host genotype, agronomic practices, weather conditions during flowering and grain fill, and post-harvest handling. Deoxynivalenol contamination reduces grain marketability and affects animal health. Reliable measurement is therefore central to grain management and trade decisions, especially during epidemic years [6,7,8,9].

A wide range of analytical approaches is available for DON determination. Laboratory-based chromatographic methods, particularly LC–MS/MS, provide high analytical specificity. They also allow DON to be measured alongside acetylated and conjugated forms [2]. These methods are resource-intensive and are not routinely available at grain elevators. As a result, commercial immunoassay-based rapid test kits, including lateral flow devices and enzyme-linked immunosorbent assays (ELISA), are widely used for on-site screening of maize deliveries.

Despite their advantages, rapid DON test kits introduce two distinct sources of variability. First, antibody-based assays may cross-react with structurally related trichothecenes and masked mycotoxins. This can influence apparent DON concentrations [10,11,12,13]. Second, sampling effects can introduce substantial variability, particularly when heterogeneous truckloads of grain are tested under commercial receiving conditions [14]. During the 2018 epidemic, these analytical and sampling-related factors were often indistinguishable in practice, contributing to inconsistent test outcomes and repeated delivery attempts by maize growers seeking a favorable result.

Comprehensive reviews of DON epidemiology and management emphasize that predictive models, agronomic interventions, and post-harvest segregation strategies all rely on accurate and interpretable measurements of DON at multiple points in the grain supply chain. In this context, Munkvold (2019) [15] emphasizes that mycotoxin biology, sampling uncertainty, and analytical performance must be considered together when evaluating risk. Clearly, confidence in DON testing reflects biological variability, sampling, and analytical measurement rather than analytical performance alone.

Previous work in Ontario has documented spatial variability in DON contamination [16,17]. Grain elevators also face practical constraints in applying rapid DON testing under commercial receiving conditions [8]. However, prior studies have typically examined analytical cross-reactivity or sampling variability in isolation. The events of 2018 demonstrated that, under epidemic conditions, these sources of variability interact in ways that directly affect commercial decision-making and stakeholder confidence.

Building on this context, this study was conducted to (i) characterize analytical cross-reactivity among commonly used DON immunoassays under controlled conditions, (ii) examine their response in naturally contaminated maize containing DON derivatives, and (iii) quantify variability in DON measurements arising from routine grain elevator testing. By distinguishing analytical specificity from sampling- and site-level effects, this work seeks to explain the sources of unreliable DON test results observed during the 2018 epidemic and to inform more transparent and defensible interpretation of rapid DON measurements in commercial maize handling systems.

## 2. Results

### 2.1. Analytical Responses of DON Immunoassays to Structurally Related Compounds

All the commercial kits accurately quantified DON at 1 mg/kg relative to LC-MS/MS under controlled spiking conditions; however, substantial differences were observed in responses to DON derivatives (Table 1). Veratox ELISA showed high cross-reactivity to 15-acetyldeoxynivalenol (15ADON) (~80%), whereas Reveal Q and QuickTox showed lower cross-reactivity (~10–11%), and Rida-Quick DON showed none. Cross-reactivity to deoxynivalenol 3-glucoside (D3G) was low in Veratox ELISA (~10%), moderate in Reveal Q (~23%), and high in QuickTox (~50%); Rida-Quick DON again showed minimal response. Kits displayed highly variable responses to 3-acetyldeoxynivalenol (3ADON), ranging from minimal to strongly positive depending on method and matrix (Table 1). Matrix effects were evident, particularly for Reveal Q and Rida-Quick DON when presented with DON + 15ADON or DON + 3ADON mixtures. For DON + D3G, all kits except QuickTox showed pronounced matrix-dependent differences. Across DON + 15ADON combinations, Veratox ELISA consistently showed high cross-reactivity, whereas Reveal Q showed the lowest. All kits except LC-MS/MS produced total DON estimates >1 mg/kg in combination-spiked samples. QuickTox showed strong cross-reactivity to DON + D3G + 3ADON mixtures in all matrices.

### 2.2. Laboratory Comparison Using Naturally Contaminated Maize

In maize containing 4.3 mg/kg DON (LC–MS/MS), immunoassay readings ranged from essentially the same as the reference (within ~2%) to about 26% above the LC–MS/MS value (Table 2). Differences were consistent with the presence of DON derivatives in the naturally contaminated material, as indicated by divergence between DON and TDON total DON; the sum of DON plus its measured acetylated and conjugated derivatives) measured by LC–MS/MS.

### 2.3. Interlaboratory Variation Among 61 Grain Elevators

**Figure 1:**
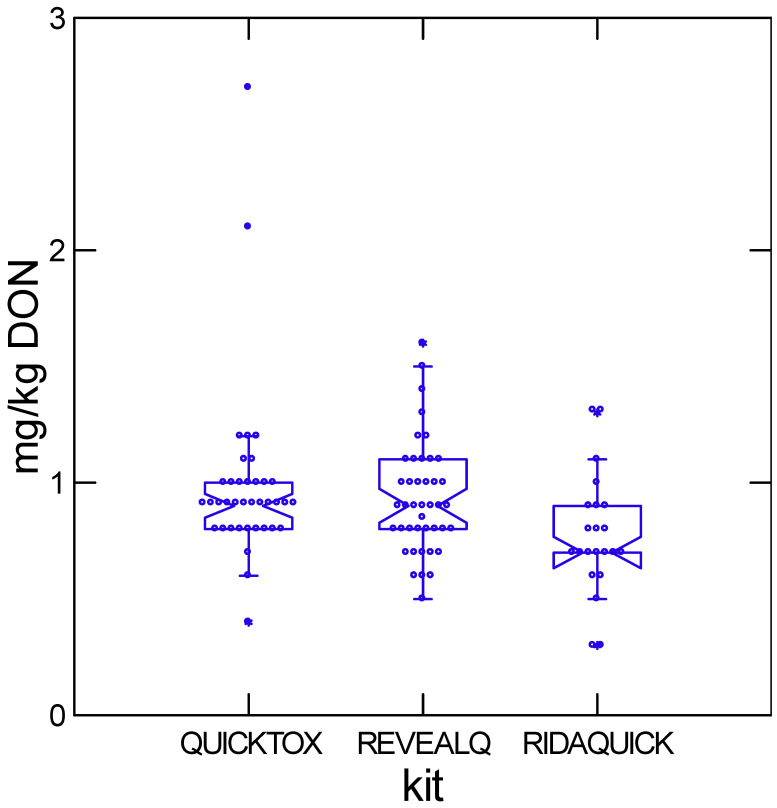
Variation in concentration reported from country elevators sent maize at 1 mg/kg DON for the most commonly used kits in Ontario. Boxes show the interquartile range with median; whiskers extend to 1.5× IQR. Asterisks indicate outliers beyond this range. Raw data for this table are available in Supplementary Information File S3.

**Figure 2:**
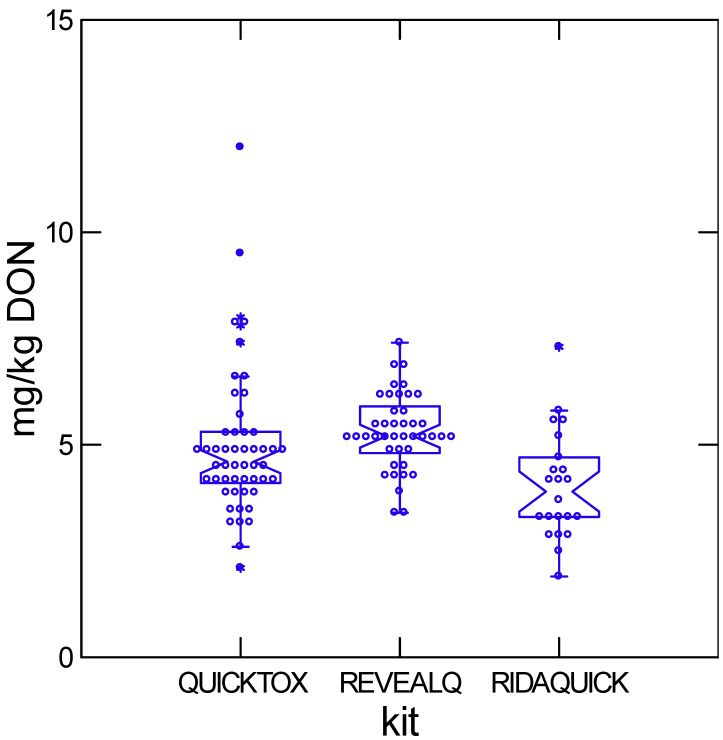
Variation in concentration reported from country elevators sent maize at 5 mg/kg DON for the most commonly used kits in Ontario. Boxes show the interquartile range with median; whiskers extend to 1.5× IQR. Asterisks indicate outliers beyond this range. Raw data for this table are available in Supplementary Information File S3.

A total of six commercial DON test kits were represented among the 61 participating grain elevators. Among the six, the top three were QuickTox, Reveal Q, and Rida-Quick DON, which accounted for approximately 41%, 34%, and 18% of reported kit usage, respectively. Performance of these three kits at nominal DON levels of approximately 1 and 5 mg/kg is shown in Figure 1 and Figure 2. At lower DON concentrations, mean values from all three kits approximate the LC-MS/MS reference (1.0 mg/kg). In contrast at higher DON concentrations, Rida-Quick DON produced significantly lower estimates than the other two kits (Table 3). Variability increased substantially at ~5 mg/kg. QuickTox exhibited the greatest deviation, with the highest proportion of extreme outliers and the lowest proportion of results within ±20% of the LC-MS/MS value. Approximately 16% of the reported results were over 20% higher than the LC–MS/MS reference, and a similar fraction were more than 20% below the reference.

### 2.4. Specificity of DON Measurement in Truckload Probe Samples

LC–MS/MS DON concentrations in 49 truckload probe samples were classified into three categories for presentation: 2.3 ± 0.35 mg/kg (low), 4.6 ± 0.76 mg/kg (intermediate), and 5.8 ± 0.94 mg/kg (high). All immunoassay kits overestimated DON at ~2 mg/kg in truckload probe samples, with QuickTox and Veratox ELISA ~83% higher than LC–MS/MS (Table 4). Accuracy improved in intermediate- and high-DON categories, where most kits matched LC–MS/MS more closely.

Results from truckload probe samples are summarized in Table 4. At low DON (~2 mg/kg), all immunoassays overestimated LC–MS/MS DON, with QuickTox and Veratox ELISA ~83% higher and Reveal Q and Rida-Quick DON 52–56% higher; accuracy improved markedly at intermediate and high concentrations where differences generally fell below 10% (Table 4). This contrasts with the interlaboratory blinded sample results in Table 3, where mean values at ~2–3 mg/kg approximated LC–MS/MS and the proportion of results within ±20% remained high, while at ~6–7 mg/kg Rida-Quick DON was closer to the reference than QuickTox, which showed greater deviation and more extreme outliers (Table 3). Together, these comparisons indicate that sampling context (truckload probes vs. blinded ground samples) amplifies positive bias at low DON, whereas kit-specific performance differences are evident across both datasets.

## 3. Discussion

Most studies evaluating antibody-based DON assays rely on spiked laboratory samples. Here, Section 2.1 and Section 2.2 examine analytical specificity under controlled conditions. Section 2.3 addresses interlaboratory variability, and Section 2.4 examines the effect of sampling context. This study extends that work by examining cross-reactivity, matrix effects, and real-world analytical variation across laboratory and grain elevator environments. In Ontario, *F. graminearum* populations are dominated by the 15ADON chemotype [17]. Naturally contaminated maize therefore contains mixtures of DON, 15ADON, and often D3G. In this study, QuickTox and Rida-Quick DON underestimated DON plus acetylated derivatives in mixtures dominated by 15ADON relative to LC–MS/MS. When DON + D3G were considered, all four kits underestimated total mycotoxin content (Table 1). Dzuman et al. reported similar variation due to matrix effects [9]. Analytical variation increased with DON concentration, consistent with Tittlemier and Whitaker [8]. At low DON (0.8 ± 0.02 mg/kg), kit estimates approximated LC–MS/MS. At high DON (4.3 ± 0.14 mg/kg), two kits remained accurate, whereas QuickTox read substantially higher, consistent with its demonstrated cross-reactivity to both D3G and 3ADON (Table 1 and Table 2). Interlaboratory testing among 61 elevators revealed substantial variability at relatively high DON concentrations of ~5 mg/kg. QuickTox produced the largest number of extreme outliers. Reveal Q and Rida-Quick showed narrower but still significant variation. These findings show that both cross-reactivity and site-to-site variation contribute to inconsistent DON measurements. Probe sample results aligned with earlier findings: all kits overestimated DON at ~2 mg/kg, whereas accuracy improved at higher concentrations. We assumed that a well-designed probe composite sample would be representative of the whole truckload and analogous to continuous flow monitoring, which is an approach supported by standard grain sampling guidelines [8]. Overall, the findings demonstrate that cross-reactivity with DON derivatives and interlaboratory variation both contribute to inconsistent DON measurement in Ontario’s grain handling system. These results support ongoing calls for standardized reference materials and blind check programs and further suggest that such reference materials be used by grain purchasers to regularly calibrate on-site DON testing protocols, thereby improving consistency and reliability across commercial testing environments [18,19].

While analytical specificity to native DON concentration is essential for regulatory compliance and commercial grain trade decisions, the optimal characteristics of a DON screening assay may depend on the intended end use of the grain. For example, in livestock production systems particularly swine, deoxynivalenol-3-glucoside (D3G) is toxicologically relevant. It can be hydrolyzed to DON during digestion [20,21,22]. In this context, immunoassays exhibiting partial cross-reactivity to D3G may provide a more conservative estimate of biologically relevant DON equivalents, whereas assays designed to minimize cross-reactivity may underestimate feeding risk when masked forms are present. Conversely, in the general grain trade where contractual limits and regulatory thresholds are defined strictly on DON concentration, higher analytical specificity to DON itself may be preferable. No single rapid test kit is universally optimal. Kit selection and interpretation should reflect the decision context and acceptable risk. Comparison of Table 3 and Table 4 shows that the sampling context strongly influences DON estimates: truckload probe samples amplified positive bias at low concentrations, whereas blinded ground samples yielded values closer to LC–MS/MS. Despite this, kit-specific trends, such as the QuickTox reading higher and Rida-Quick DON closer to reference at high DON, were consistent across both datasets. This reinforces the need for harmonized protocols and certified reference materials.

## 4. Conclusions

This study demonstrates that variability in DON immunoassay performance arises from two major factors: cross-reactivity with DON derivatives and differences in sampling context. Commercial Veratox ELISA and lateral flow kits showed substantial variation in sensitivity to 15ADON, 3ADON, and D3G. These differences, combined with matrix effects, influence accuracy. Real-world testing revealed that interlaboratory variation and sampling method strongly affect DON estimates, with systematic overestimation observed in truckload probe samples at low concentrations and improved alignment at higher concentrations. These findings support the need for harmonized testing protocols, blind check programs and certified reference materials to ensure reliable DON assessment. Accurate measurement therefore reduces economic risk and improves food and feed safety.

## 5. Materials and Methods

### 5.1. Statistical Analysis

All statistical analyses were conducted using SAS software (version 9.4; SAS Institute Inc., Cary, NC, USA) [23]. Completely randomized designs and factorial arrangements were analyzed using analysis of variance (ANOVA) for testing analytical methods, DON level or category, and their interactions specified as fixed effects, as appropriate for each experiment. Residuals were examined to confirm normality and homogeneity of variance [23]. When significant main effects or interactions were detected, means were separated using the Tukey–Kramer multiple comparison procedure with a significance level of *p* ≤ 0.05.

### 5.2. Quality Control

Quality control procedures were applied throughout sample preparation and analysis [15]. Certified reference materials, calibration standards, blanks and replicate samples were included to monitor analytical performance. Acceptance criteria for recoveries, precision, and analytical variation followed established procedures described for DON analysis in maize, including the use of ±20% agreement thresholds for evaluating consistency with reference measurements.

### 5.3. Immunoassay Responses to DON and Related Compounds Under Controlled Spiking Conditions

Responses of commercial kits to DON, 3ADON, 15ADON, and D3G were evaluated in water and maize matrices. Mycotoxin standards (>98% purity) were obtained from Biopure (Romer Labs). DON and 15ADON were supplied as solids; 3ADON and D3G were supplied as solutions. Except for D3G, standards were reconstituted in acetonitrile. Finely ground maize (<3.12 ng/g DON) was used to prepare 10 g matrices in 50 mL Falcon tubes. Pure toxins were added to achieve 1 mg/kg individually and in combinations.

Samples were dried overnight at room temperature (approximately 22 °C) in a chemical fume hood with ambient airflow to allow complete evaporation of solvent prior to extraction. For immunoassay analysis, dried samples were extracted using the extraction solvents, solvent volumes, and extraction procedures specified by each kit manufacturer. Briefly, 8 g subsamples were combined with the appropriate extraction solvent at the manufacturer-recommended solvent-to-sample ratio, shaken mechanically for the prescribed duration, and allowed to settle prior to analysis. Extracts were clarified as required by the individual test protocols before application to lateral flow devices or ELISA plates.

Subsamples (2 g) were analyzed by LC–MS/MS (Agilent 1100 Series HPLC unit with G1316A thermostat column oven, G1312A binary pump and G1322A degasser, Agilent Technologies, Santa Clara, CA, USA) attached to a HTC Pal autosampler (CTC Analytics, Zwingen, Switzerland) equipped with a 100-μL injection syringe. Mass spectrometry was performed using a PE Sciex API 365 triple quadrupole mass spectrometer (AB SCIEX, Concord, ON, Canada) modified with an IONICS EP10+ detector upgrade (Concord, ON, Canada) equipped with an electrospray ionization source (ESI, Sciex TurboIonspray Concord, ON, Canada) [16]. Chromatographic separation with a reverse-phase C18 column (2.1 × 100 mm, 1.7 µm particle size) (ZORBAX Eclipse Plus Part Number: 959758-902, Agilent Technologies, Santa Clara, CA, USA), maintained at 40 °C, was used. Mobile phase A consisted of water with 0.1% formic acid and mobile phase B consisted of methanol with 0.1% formic acid. A linear gradient was applied from 5% to 95% B over 10 min at a flow rate of 0.3 mL/min, followed by re-equilibration. The injection volume was 5 µL. A triple-quadrupole mass spectrometer equipped with an electrospray ionization source operating in negative ion mode was used for detection. Quantification was based on external calibration using matrix-matched standards. This configuration seemed to give a better separation of 3ADON and 15ADON.

Limits of detection and quantification for DON were 0.05 mg/kg and 0.15 mg/kg, respectively. Calibration curves consisted of at least five concentration levels spanning the analytical range, with acceptable linearity (R^2^ ≥ 0.99). All extractions were replicated four times. A completely randomized design (CRD) was used with analytical method as a fixed effect.

### 5.4. Laboratory Comparison Using Naturally Contaminated Maize

Two groups of maize samples with DON concentrations near 1 mg/kg and 5 mg/kg were prepared for analysis. Each sample within each group was first coarsely milled using a No. 60 Power Grist Mill (The C.S. Bell Co., Tiffin, OH, USA) thoroughly mixed and then subjected to fine grinding using a Steinlite Mill (Seedburo Equipment Co. Des Plaines, IL USA) with a 1 mm screen. Coarse grinding was performed using the manufacturer’s standard screen, followed by thorough manual mixing. Samples were passed once through each mill, with mills cleaned between samples to prevent cross-contamination. Following fine grinding, each sample was manually homogenized to ensure uniform particle distribution prior to subsampling.

Samples (10 g or 50 g) were analyzed using QuickTox™, Reveal^®^Q+, Veratox^®^ELISA, and Rida^®^Quick DON according to manufacturer instructions. Extraction and immunoassay procedures were performed as described in Section 5.3. LC–MS/MS served as the reference method and was conducted as described in Section 5.3. A 2 × 5 factorial CRD was used with DON level and analytical method as fixed effects.

### 5.5. Interlaboratory Variation Among Grain Elevator Testing Sites

Naturally contaminated maize used for the interlaboratory comparison was sourced from a bulk lot identified to contain elevated deoxynivalenol concentrations during the 2018 growing season in Ontario. The grain was transported to the laboratory, thoroughly mixed, and processed to produce a uniform test material. The bulk lot was first coarsely ground and homogenized, after which the material was finely ground to ensure uniform particle size and mycotoxin distribution. The finely ground maize was mixed extensively prior to subsampling and analyzed by LC–MS/MS to establish approximate DON concentrations for low- and high-test materials.

Standardized 50 g aliquots of the homogenized maize were weighed and packaged into coded envelopes to create blinded test samples. A total of four blinded samples (two low and two high DON concentrations) were prepared for each participating site. Envelopes were shipped to 61 grain elevators. Elevator staff analyzed samples using their routine DON test kits under normal operating conditions, without harmonization of extraction procedures, calibration practices, or decision thresholds. The top 3 most frequently used kits were used in this analysis. LC–MS/MS reference analysis was conducted as described in Section 5.3.

### 5.6. Specificity of DON Measurement in Truckload Probe Samples

Probe samples (approximately 2 kg) were collected from 49 truckloads of maize at the Independent Grain Processing Cooperative (IGPC) using a mechanical compartmental grain probe designed for truckload sampling. For each truck, the probe was inserted at multiple locations distributed across the load to capture spatial heterogeneity, with individual probe increments combined to form a composite sample. Composite probe samples were mixed thoroughly immediately after collection.

Samples were transported to the laboratory on the day of collection and processed without extended storage. Samples were ground and homogenized as described in Section 5.4. From the homogenized material, analytical subsamples (10 g) were obtained for immunoassay and LC–MS/MS analysis.

Subsamples were analyzed by Reveal Q, QuickTox, Rida-Quick DON, and Veratox ELISA according to manufacturer instructions. LC–MS/MS reference analysis was conducted as described in Section 5.3. Based on LC–MS/MS DON concentrations, samples were grouped post hoc into lower (<3 mg/kg), intermediate (4–5 mg/kg), and higher (>6 mg/kg) DON categories for data presentation. A 3 × 6 factorial completely randomized design was used with DON category and analytical method as fixed effects.

## Figures and Tables

**Table 1:** Variance analysis of DON concentrations measured in maize and water matrices following spiking with DON or related compounds, as determined by immunoassays and LC–MS/MS.

	^c^ DON	^c^ D3G	^c^ 15ADON	^c^ 3ADON
Effect	*p*-Value	*p*-Value	*p*-Value	*p*-Value
Matrix (M)	0.1138	0.5506	0.1663	0.3263
Analytical Method (AM)	0.0592	<0.0001	<0.0001	<0.0001
M x AM	0.3488	0.7243	0.6685	0.0206
	**^b^ Measured**	**^b^ Measured**	**^b^ Measured**	**^b^ Measured**
**^a^ Analytical Method**	**DON**	**D3G**	**15ADON**	**3ADON**
LC-MS/MS	0.95 ± 0.020a	0.95 ± 0.069a	1.01 ± 0.079a	1.02 ± 0.044c
Veratox ELISA	0.97 ± 0.021a	0.10 ± 0.007d	0.76 ± 0.060a	0.11 ± 0.004d
Reveal Q	0.95 ± 0.020a	0.23 ± 0.016c	0.11 ± 0.010b	0.87 ± 0.038c
QuickTox	1.02 ± 0.022a	0.49 ± 0.035b	0.10 ± 0.016b	1.34 ± 0.058b
Rida-Quick	0.95 ± 0.020a	0.00 ± 0.000e	0.00 ± 0.000c	1.73 ± 0.076a

^a^ Method; QuickTox (EnviroLogix Inc., Portland, ME, USA), Reveal Q (Neogen Corp., Lansing, MI, USA) and Rida-Quick. (R-Biopharm Inc., AG Darmstadt, Germany) compared to Veratox ELISA (Diagnostix Ltd., Mississauga, ON, Canada) and validated by LC-MS/MS. ^b^ Concentrations are reported as mean ± standard error (mg/kg). Concentrations sharing a common letter do not differ at α = 0.05 (Tukey–Kramer adjustment). ^c^ Abbreviations: DON, deoxynivalenol; D3G, deoxynivalenol-3-glucoside; 15ADON, 15-acetyldeoxynivalenol; 3ADON, 3-acetyldeoxynivalenol. Raw data for this table are available in Supplementary Information File S1.

**Table 2:** Measured DON and total DON concentrations in naturally contaminated maize samples with relatively lower and higher DON as obtained using immunoassays and LC–MS/MS.

^a^ DON Concentration Category	^b^ Analytical Method	^c^ Measured DON	Difference vs. LC-MS/MS DON (%)	Difference vs. LC-MS/MS ^d^ TDON (%)
Lower DON	QuickTox	1.0 ± 0.03e	25	9
	Veratox ELISA	0.8 ± 0.02f	0	27
	Reveal Q	0.8 ± 0.02f	0	27
	Rida-Quick (DON)	1.0 ± 0.03e	25	9
	LC-MS/MS (DON)	0.8 ± 0.02f		
	LC-MS/MS (TDON)	1.1 ± 0.03e		
Higher DON	QuickTox	5.4 ± 0.17ab	26	13
	Veratox ELISA	4.8 ± 0.15bc	12	23
	Reveal Q	4.6 ± 0.15cd	7	26
	Rida-Quick (DON)	4.2 ± 0.13d	2	32
	LC-MS/MS (DON)	4.3 ± 0.14cd		
	LC-MS/MS (TDON)	6.2 ± 0.20a		

^a^ Approximate concentrations of DON in maize sample categories were ~1 mg/kg (for lower) and ~5 mg/kg (for higher). ^b^ Method; QuickTox (EnviroLogix Inc.), Reveal Q (Neogen Corp.) and Rida-Quick (R-Biopharm Inc.) compared to Veratox ELISA (Diagnostix Ltd.) and validated by LC-MS/MS. ^c^ DON concentrations are reported as mean ± standard error (mg/kg). Concentrations sharing a common letter do not differ at α = 0.05 (Tukey–Kramer adjustment). ^d^ TDON, total DON; the combined concentration of DON and its measured derivatives (D3G, 15ADON, and 3ADON). Raw data for this table are available in Supplementary Information File S2.

**Table 3:** DON concentrations in lower- and higher-DON maize grain samples as reported by three commercial test kits at grain elevators, with the proportion of sites reporting results within ±20% of the reference sample.

DON Concentration Category ^a^	Analytical Method ^b^	Measured DON ^c^	Sites Within ±20% of Reference Sample (%) ^d^
Lower DON	QuickTox	1.0	80
	Reveal Q	0.9	71
	Rida-Quick DON	0.8	55
Higher DON	QuickTox	5.0	68
	Reveal Q	5.3	91
	Rida-Quick DON	4.1	91

^a^ Approximate concentrations of DON in maize sample categories were ~1 mg/kg (for lower) and ~5 mg/kg (for higher). ^b^ QuickTox (EnviroLogix Inc.), Reveal Q (Neogen Corp.) and Rida-Quick (R-Biopharm Inc.). ^c^ Mean DON concentrations as reported (mg/kg). ^d^ Percentage of elevator sites reporting values within ±20% of the LC-MS/MS reference DON concentration. Raw data for this table are available in Supplementary Information File S3.

**Table 4:** DON concentrations measured in truckload probe samples using commercial immunoassays, with comparison to LC–MS/MS DON and total DON reference values.

^a^ DON Concentration Category	^b^ Method	^c^ Measured DON	Difference vs. LC-MS/MS DON (%)	Difference vs. LC-MS/MS TDON (%)
Lower DON	Reveal Q	3.5 ± 0.55c	52	35
	QuickTox	4.2 ± 0.66bc	83	62
	Rida-Quick DON	3.6 ± 0.55c	56	38
	Veratox ELISA	4.2 ± 0.65bc	83	62
	LC-MS/MS (DON)	2.3 ± 0.35d		
	^d^ LC-MS/MS (TDON)	2.6 ± 0.41d		

Intermediate DON	Reveal Q	4.3 ± 0.67bc	7	19
	QuickTox	4.2 ± 0.69bc	9	21
	Rida-Quick DON	4.5 ± 0.70bc	2	15
	Veratox ELISA	5.0 ± 0.80abc	9	6
	LC-MS/MS (DON)	4.6 ± 0.76bc		
	LC-MS/MS (TDON)	5.3 ± 0.87abc		

Higher DON	Reveal Q	5.5 ± 0.90abc	5	26
	QuickTox	6.9 ± 1.11a	19	7
	Rida-Quick DON	6.3 ± 1.02ab	9	15
	Veratox ELISA	7.6 ± 1.21a	31	3
	LC-MS/MS (DON)	5.8 ± 0.94abc		
	LC-MS/MS (TDON)	7.4 ± 1.18a		

^a^ Approximate concentrations of DON in maize sample categories were ~2–3 mg/kg (for lower), ~4–5 mg/kg (for intermediate) and ~6–8 mg/kg (for higher). ^b^ Method; QuickTox (EnviroLogix Inc.), Reveal Q (Neogen Corp.) and Rida-Quick (R-Biopharm Inc.) compared to Veratox ELISA (Diagnostix Ltd.) and validated by LC-MS/MS. ^c^ DON concentrations are mean ± standard error (mg/kg). Concentrations sharing a common letter do not differ at α = 0.05 (Tukey–Kramer adjustment). ^d^ TDON, total DON; the combined concentration of DON and its measured derivatives (D3G, 15ADON, and 3ADON). Raw data for this table are available in Supplementary Information File S4.

## Data Availability

The original contributions presented in this study are included in the article/Supplementary Materials. Further inquiries can be directed to the corresponding author(s).

## Supplementary Materials

The following supporting information can be downloaded at: https://www.mdpi.com/article/10.3390/toxins18020081/s1, File S1: Analytical Responses of DON Immunoassays to Structurally Related Compounds.xlsx, File S2: Laboratory Comparison Using Naturally Contaminated Maize.xlsx, File S3: Interlaboratory Variation Among Grain Elevator Testing Sites.xlsx, File S4: Specificity of DON Measurement in Truckload Probe Samples.xlsx.

## Abbreviations

The following abbreviations are used in this manuscript:

DON	Deoxynivalenol
3ADON	3-acetyl-deoxynivalenol
15ADON	15-acetyl-deoxynivalenol
D3G	deoxynivalenol 3-glucoside
TDON	Total DON (DON + 3ADON + 15ADON + D3G)
LC-MS/MS	Liquid Chromatography–Tandem Mass Spectrometry
ELISA	Enzyme-Linked Immunosorbent Assay
LFD	Lateral Flow Device
CRD	Completely Randomized Design

## References

[1] Bianchini A., Horsley R., Jack M.M., Kobielush B., Ryu D., Tittlemier S., Wilson W.W., Abbas H.K., Abel S.A., Harrison G. (2015). DON occurrence in grains: A North American perspective. Cereal Foods World.

[2] Blackwell B.A., Schneiderman D., Thapa I., Bosnich W., Pimentel K., Kebede A., Harris L.J. (2022). Assessment of deoxynivalenol and derivatives in *Fusarium graminearum*-inoculated maize inbreds. Can. J. Plant Pathol..

[3] Yoshizawa T., Morooka N. (1975). Biological modification of trichothecenes by *Fusarium* spp. Appl. Microbiol..

[4] Miller J.D., Taylor A., Greenhalgh R. (1983). Production of deoxynivalenol and related compounds in liquid culture. Can. J. Microbiol..

[5] Miller J.D., Arnison P.G. (1986). Degradation of deoxynivalenol by suspension cultures of Frontana wheat. Can. J. Plant Pathol..

[6] Schaafsma A.W., Hooker D.C. (2007). Climatic models to predict occurrence of *Fusarium* toxins in wheat and maize. Int. J. Food Microbiol..

[7] Eli K., Schaafsma A.W., Hooker D.C. (2022). Impact of agronomic practices on *Fusarium* mycotoxin accumulation in maize grain. World Mycotoxin J..

[8] Tittlemier S.A., Whitaker T.B. (2023). Current sampling plans can introduce high variance in mycotoxin testing results as demonstrated by the FAO Mycotoxin Sampling Tool. World Mycotoxin J..

[9] Dzuman Z., Zachariasova M., Vaclavikova M., Fenclova M., Veprikova Z., Hajslova J. (2014). Investigation of the impact of sample matrix on accuracy of deoxynivalenol ELISA. Anal. Bioanal. Chem..

[10] Tangni E.K., Motte J.C., Callebaut A., Pussemier L. (2010). Cross-reactivity of antibodies in commercial deoxynivalenol test kits against fusariotoxins. J. Agric. Food Chem..

[11] Ruprich J., Ostry V. (2008). Cross-reactivity of antibodies against deoxynivalenol with deoxynivalenol-3-glucoside. Cent. Eur. J. Public Health.

[12] Gonçalves C., Stroka J. (2016). Cross-reactivity features of deoxynivalenol-targeted immunoaffinity columns. Food Addit. Contam. A.

[13] Uhlig S., Stanic A., Hussain F., Miles C.O. (2017). Selectivity of commercial immunoaffinity columns for modified forms of deoxynivalenol. J. Chromatogr. B.

[14] Tittlemier S.A., Gaba D., Chan J.M. (2013). Monitoring of trichothecenes in Canadian cereal shipments. J. Agric. Food Chem..

[15] Munkvold G.P. (2019). Crop management practices to minimize the risk of mycotoxin contamination in maize. Toxins.

[16] Limay-Rios V., Schaafsma A.W. (2021). Relationship between mycotoxin content in winter wheat grain and aspirated dust. ACS Omega.

[17] Crippin T., Limay-Rios V., Renaud J.B., Schaafsma A.W., Sumarah M.W., Miller J.D. (2020). *Fusarium graminearum* populations from corn and wheat in Ontario, Canada. World Mycotoxin J..

[18] Gilbert J., Startin J.R., Sharman M., Crews C., Massey R., MacDonald S., Parker I. (1992). Deoxynivalenol in wheat and maize flour reference materials. Food Addit. Contam..

[19] Tittlemier S.A., Brunkhorst J., Chan J.M. (2025). Design of a quality control scheme to assess sample preparation performance for deoxynivalenol in wheat. World Mycotoxin J..

[20] Berthiller F., Dall’Asta C., Schuhmacher R., Lemmens M., Adam G., Krska R. (2005). Masked mycotoxins: Determination of a deoxynivalenol glucoside in artificially and naturally contaminated wheat by liquid chromatography–tandem mass spectrometry. J. Agric. Food Chem..

[21] Berthiller F., Crews C., Dall’Asta C., De Saeger S., Haesaert G., Karlovsky P., Oswald I.P., Seefelder W., Speijers G., Stroka J. (2013). Masked mycotoxins: A review. Mol. Nutr. Food Res..

[22] Nagl V., Woechtl B., Schwartz-Zimmermann H.E., Hennig-Pauka I., Moll W.-D., Adam G., Berthiller F. (2014). Metabolism of the masked mycotoxin deoxynivalenol-3-glucoside in pigs. Toxicol. Lett..

[23] Bowley S. (2015). A Hitchhiker’s Guide to Statistics in Plant Biology—GLMM Edition.

